# Determinants of virologic failure among adult HIV patients on first line antiretroviral treatment in Oromia, Central Ethiopia: 2022 a case-control study

**DOI:** 10.1186/s12981-024-00625-4

**Published:** 2024-06-24

**Authors:** Worku Gidisa Ayana, Mulatu Ayana Hordofa, Abebe Dechasa Yadeta

**Affiliations:** 1 HIV Case Detection, Treatment and Care, Woliso Health Center, Waliso, Ethiopia; 2 Department of Public Health, College of Medicine and Health Science, Ambo University, Ambo, Ethiopia; 3 Department of Nursing, College of Medicine and Health Science, Ambo University, Ambo, Ethiopia

**Keywords:** Adults, Determinants, Failure, HIV, Clients

## Abstract

**Background:**

Ethiopia’s viral suppression rate was less than 90% by 2020, and more than 10% of adult clients on ART in Woliso Town were unsuppressed at the end of March 2022. This study aims to identify determinants of virologic failure among adult clients on ART at health facilities in Oromia region of Ethiopia.

**Methods:**

A facility-based unmatched case-control study was conducted at health facilities in Oromia region from August 1 to September 1, 2022. The study cases were clients with virologic-confirmed first-line ART failure, while controls were clients on first-line ART with a suppressed viral load. A total of 135 cases and 268 control participants were selected using simple random sampling techniques, and data were collected by reviewing the client’s document. Epi-Info7 was used for data entry and SPSS version 20 for data analysis. Variables having a P-value of less than 0.25 in the bi-variable analysis were included in multivariable logistic regression. Determinants of virologic failure were determined based on an adjusted odds ratio using 95% CI and a P-value of < 0.05.

**Result:**

In this study, clients with an age ≥ 35 years (AOR = 3.4, 95% CI: 1.6, 7.0), clients with a baseline regimen of AZT + 3TC + NVP (AOR = 3.5, 95% CI: 1.4, 8.8), clients with a base-line CD4 count < 350 mm^3^ (AOR = 2.3, 95% CI: 1.1, 4.5), being single marital status (AOR = 3.7, 95% CI: 1.4, 10.5), TB-HIV coinfection (AOR = 2.58, 95% CI: 1.3, 5.1), and having opportunistic infection other than TB in the last six months (AOR = 3.06, 95% CI: 1.5, 6.3) were factors significantly associated with virologic failure while clients within the appointment spacing model (AOR = 0.05, 95% CI: 0.03, 0.10) is inversely associated with virologic failure.

**Conclusion:**

This study showed that age ≥ 35 years, being single, baseline ART regimen with (AZT + 3TC + NVP), baseline CD4 cell count < 350 mm^3^, Tb-co infection, and opportunistic infection in the last 6 months were factors associated with virologic failure. Involvement in the appointment spacing model was found to be protective.

## Introduction

HIV Virologic failure among People living with HIV is steadily growing across all countries in the world. Virologic failure is defined as a persistently detectable viral load over 1000 copies/mL (that is, two consecutive viral load assessments within a 3-month interval with adherence assistance in between measurements). According to the WHO guideline to detect and confirm treatment failure and to switch to another ART regimen, viral load is indicated as the preferable monitoring method (strong recommendation) [1]. 

Although viral suppression varies by region, Ethiopia’s viral suppression rate was less than 90% by 2020 [2]. As of March 2022, a report from St. Luke Hospital and Woliso Health Centre shows that of a total of 2522 clients having follow-up for HIV care and treatment, 264 (10.5%) were on second-line ART. This is very high when compared with the standard 5% expected [3]

Although different initiatives were implemented for HIV prevention and control, virologic failure continued and became a major cause of morbidity and mortality among HIV-positive clients in the study area. This research is aimed at identifying determinants of virologic failure among patients attending treatment at health facilities in Woliso town, Oromia region, Ethiopia.

The finding of this research may help local HIV program implementers to give their attention to how to prevent virologic failure among first-line ART clients. It also can help HIV Program managers to control drug resistant HIV disease and to attain the third 95’s target set by WHO to be achieved by 2030. The community will be protected from drug-resistant HIV disease and AIDS-related morbidity and mortality may be minimized. Finally, other researchers can use this finding to study more.

## Methods

### Study design and study area

A facility-based unmatched case-control study was conducted at Woliso Town public health facilities in the Oromia region of Ethiopia. Woliso town is located 114 km from the capital city, Addis Ababa, to the southwest. The two health facilities, St. Luke Hospital and Woliso Health Centre are found in the town. As of July 2022, the hospital was providing ART to 1543 adult HIV-positive clients. Of those, 220 were receiving second-line ART, while 1323 were receiving first-line ART. As of July 2022, the health center had 886 adult clients on ART; of this, 44 were on the second-line regimen and, the rest, 842, were on the first-line regimen. Data was collected from August 1 to September 1, 2022.

### Source population

The source populations of the cases were all clients who were on ART and who had virologic-confirmed first-line antiretroviral therapy failure. All clients who were on ART, who did not experience virologic failure, and who were receiving first-line antiretroviral medication at both St. Luke Hospital and Woliso Health Centre were included as sources of control.

### Study population

The study population for cases was all randomly selected HIV-positive adult clients who were on ART at both St. Luke Hospital and Woliso Health Centre and were confirmed to have failed virologic therapy following at least 6 months of first-line ART therapy. The study populations for controls were randomly selected HIV-positive adult clients who were on ART at both St. Luke Hospital and Woliso Health Centre who had suppressed the virus and were on first-line ART treatment after at least 6 months on first-line ART.

### Inclusion and exclusion criteria

Inclusion criteria for cases: Adult clients on antiretroviral treatments that had confirmed virologic failure after at least 6 months on first-line ART. Inclusion criteria for controls: Adult clients on antiretroviral treatment who had suppressed viral load (plasma viral load of < 1000 copies/ml) and on first-line ARV following at least 6 months of first-line ART therapy. Exclusion criteria: clients who have no viral load result; clients with incomplete variables were excluded from both cases and controls.

### Sample size determination

The sample size was determined using Epi Info version 7 by taking into account the determining factor of virologic failure taken from different research conducted on similar topics. The significant determinant factor of virologic failure variables, which gives the highest sample size, was used. From the determinant variables, baseline WHO clinical stages III and IV were the ones that resulted in the largest sample size. The calculated sample size was 365(122 cases and 243 controls) based on a 5% margin of error and 80% power. Since a case is relatively small compared to a control, a case-to-control ratio of 1:2 is used, the percent of control exposed is 81.5%, and the adjusted odds ratio is 2.9 [4]. Since the calculated sample size is 365 (122 cases and 243 controls), by adding 10% non-response, the final sample size becomes 403 (135 cases and 268 controls).

### Sampling technique

St. Luke Hospital has 1271 adults on first-line ART with registered viral load results, and 220 adults were on second-line treatment. From the total number of clients on the second line, 186 had virologic-confirmed treatment failure; those stayed for more than six months on the first line ART. Similarly, at Woliso Health Centre, of the 886 adults on ART, 803 were adult clients on the first line of ART who stayed for more than six months. Of 803 clients, 796 are on first-line ART with viral load results. Out of 44 clients on second-line treatment, 36 had virologic-confirmed first-line ART failure.

So, when the proportional allocation was applied at both health facilities, since the total sample size calculated was 135 cases and 268 controls, including 10% added for non-response, 22 cases and 105 controls were selected from Woliso Health Centre, and 113 cases and 163 controls were selected from St. Luke Hospital. A simple random sampling approach was used in the sampling operation for both cases and controls. A medical record number from the routine viral load register was used as a sampling frame for both cases and controls.

### Operational definition of variables

**Virologic failure**: Viral load above 1000 copies/ml based on two consecutive viral load measurements in three months apart, with adherence support following the first viral load test as per national guidelines for individuals after at least 6 months of a new ART regimen [5]. **Adult**: Adults are defined as those ages of ≥ 15 years old in this study [5]. **Good adherence**: >95%(of 30 doses ≤ 2 doses missed), **Fair adherence**: 85-95%(of 30 dose 3–5 doses missed), **Poor adherence**: < 85%(of 30 doses ≥ 6 doses missed) [5]. **Treatment interruption**: Clients has missed appointment for greater than 28 days from the last appointment date [6]. **Drug side effect**: Is any unintended effect occurring at doses normally used in human [6]. Appointment spacing Model: Refers to patients who are deemed stable and scheduled for clinical visits and prescription refills every six months.

### Data collection tools and techniques

Data collection tools were developed from the Ethiopian national consolidated ART guidelines, the client’s intake form, the client information sheet, and the high viral load register. Client’s follow-up card, adherence supporters register, appointment spacing model register, and also some questions were from previous research conducted [7]. Data were extracted from the client’s folder and registered using a pretested, structured checklist. The checklist was divided into four sessions: sociodemographic factors, treatment adherence status, clinical-related factors, and treatment-related factors. Four health workers were trained for two days and collected the data depending on the client’s medical record number, which was selected by computer during a simple random sampling technique. The researcher and one trained supervisor closely monitoring the whole data collection process on a daily basis.

### Data quality and management

A data collection checklist pretest was conducted, and the results showed that the checklist was organized well to collect clients’ data easily from the register. Training was given to data collectors and supervisors for one day. The supervisor and researcher monitored all data collection processes and completeness daily. Data collectors collect data from clients’ folders and registers according to a predetermined client’s medical record number, which is selected by computer during simple random sampling. Before analyzing the data, it was checked and cleaned.

### Data analysis

For analysis, the collected data were coded and put into epi-info version 7 before being exported to the statistical package for social sciences (SPSS) version 20. The data were described using frequencies and proportions. To summarize descriptive data in each category, cross-tabulation was employed. To investigate factors determining virologic failure, bi-variable and multivariable logistic regression analyses were employed. Variables having a P-value of less than 0.25 in bi-variable logistic regression analyses were included in multivariable logistic regression. The model’s fitness was assessed using the Hosmer and Leme-Show goodness of fit test with a p-value greater than 0.05. Multicollinearity analysis was conducted depending on the variance inflation factor (VIF) > 5. Then, based on the findings, factors that have a significant relationship with virologic failure among first-line antiretroviral therapy were identified and reported based on the adjusted odds ratio (AOR) with a 95% confidence interval (CI) at a P-value of < 0.05.

### Ethical and legal considerations

After a written ethical clearance letter was obtained from the ethical review committee of Ambo University College of Medical and Health Sciences, a letter of permission was obtained from St. Luke Hospital management and Woliso Health Centre management for data collection. The collected data was kept locked and strictly confidential and no client-identifying information was included in the data-gathering instrument.

## Results

### Socio-demographic characteristics of the participants

A total of 403 (135 cases and 268 controls) HIV-positive clients on first-line ART were included in the study. The mean age of participants just during the last viral load test was 38 years. In the study, 72 (53.30%) of males and 63 (46.70%) of females were cases, while 165 (61.60%) of females and 103 (38.40%) of males were controls. Similarly, 41 (30.4%) of cases and 106 (39.5%) of controls were less than 35 years old, while 94 (69.6%) of cases and 162 (60.5%) of controls were greater than 35 years old. The study result showed that 66 (48.5%) of cases and 140 (52.2%) of controls were married, and 28 (20.70%) of cases and 18 (6.70%) of controls had single marital status.

### Drug-related client characteristics

The result of the study showed that 30 (22.2%) of cases and 63 (23.5%) of controls were on the d4T-3TC-NVP regimen as baseline treatment. Concerning ART regimen, just during the last viral load test, 14 (10.40%) of cases and 263 (98.1%) of controls were on a new ART regimen combination, which is DTG, whereas 121 (89.6%) of cases and 5 (1.9%) of controls were on a non-DTG-based ART regimen. From the respondents, 13 (9.6%) cases and 9 (3.3%) controls have a history of drug side effects.

### Clinical-related client characteristic

The result of baseline CD_4_ counts during ART initiation shows ^that 109 (80.7%)^ of cases and 163 (60.7%) of controls have a CD_4_ count < 350 cells/mm^3^. 55 (40.7%) of cases and 45 (16.80%) of controls have a history of TB co-infection. Of all the respondents, 26 (19.30%) of cases and 203 (75.70%) of controls have a history of ASM. Similarly, 40 (29.60%) of cases and 45 (16.80%) of controls had a BMI < 18.5 kg/m2 during the last viral load test. Regarding the baseline WHO clinical stage, 26 (19.30%) of cases and 87 (32.5%) of controls were stage one, and 47 (34.8%) of cases and 75 (28.0%) of controls were stage two, while the rest were either stage three or four. 41 (30.40%) of cases and 33 (12.30%) have had a history of an OI other than TB in the last 6 months.

### Treatment adherence status of the clients

In this study, 17 (12.6%) of cases and 34 (12.60%) of controls had poor treatment adherence. Regarding the history of treatment interruption, 44 (32.6%) of cases and 55 (20.50%) of controls had treatment interruptions during first-line ART.

### Personal and other factors

The result showed that 37 (27.4%) cases and 44 (16.4%) controls had no HIV status disclosure to their families. Similarly, 45 (33.3%) of sexual partners of cases, and 98 (36.6%) of sexual partners of control were HIV positive, and the rest were either HIV negative or unknown.

### Bi-variable logistic regression analysis

Bi-variable logistic regression analysis was used to identify candidate variables for multivariable logistic regression analysis at a p-value of < 0.25 in the bi-variable logistic regression model. The model’s fitness was assessed using the Hosmer and Leme-Show goodness of fit test, depending on a p-value greater than 0.05, and the result was 0.311, which shows the model was fit. Multicollinearity analysis was conducted depending on variance inflation factor (VIF) > 5 and found between 1.06 and 1.56, which shows no variables had a VIF > 5. The study showed that, from all variables, sex, age category, education level, marital status, baseline ART, drug side effects, baseline CD4, TB co-infection, baseline WHO clinical stage, history of ASM, current BMI, comorbidity, opportunistic infection in the last 6 months, treatment interruption, HIV disclosure status, and partner HIV status of the study participants were selected as the candidates for multivariable logistic regression analysis depending on their analysis result with a P-value < 0.25 in binary logistic regression.

### Determinants of virologic failure among HIV-positive clients on first-line ART

In multivariable logistic regression analysis, the age of participants during viral load test ≥ 35 Years, being single in marital status, AZT + 3TC + NVP regimen as baseline ART, Base-line CD4 < 350 mm^3^, having a history of TB co-infection, and having opportunistic infection other than TB in last 6 months were significantly associated with Virologic failure and having a history of ASM were inversely associated with Virologic failure (Table 1).

**Table 1:** Multivariable logistic regression analysis of determinants of Virologic failure among HIV-positive clients on first-line ART at health facilities in Woliso Town, 2022

Variables	Variable category	Category		COR(95% CI)	AOR(95% CI)	P-Value
		Cases (%)	Controls (%)			
Age	< 35 Years	41(30.4)	106(39.5)	1	1	
	≥ 35 Years	94(69.6)	162(60.5)	1.50(0.97,2.34)	3.40(1.65,7.02)	< 0.001*
Marital status	Married	66(48.9)	140(52.2)	1	1	
	Single	28(20.7)	18(6.7)	3.32(1.72,6.43)	3.77(1.35,10.51	0.011*
	Divorced & Separated	24(17.8)	61(22.8)	0.83(0.47,1.44)	0.78(0.36,1.70)	0.527
	Widowed	17(12.6)	49(18.3)	0.74(0.4,1.38)	0.44(0.18,1.12)	0.085
Baseline ART regimen	TDF + 3TC + DTG	7(5.2)	42(15.7)	0.36(0.14,0.88)	0.36(0.12,1.14)	0.081
	TDF + 3TC + EFV	45(33.3)	101(37.7)	0.94(0.54,1.64)	1.93(0.86,4.32)	0.109
	TDF + 3TC + NVP & AZT + 3TC + EFV	17(12.6)	32(11.9)	1.13(0.55,2.36)	2.05(0.75,5.62)	0.161
	AZT + 3TC + NVP	36(26.7)	30(11.2)	2.56(1.34,4.91)	3.45(1.35,8.83)	0.010*
	d4T-3TC-NVP	30(22.2)	63(23.5)	1	1	
Base-line CD4	CD4 < 350 mm^3^	109(80.7)	163(60.8)	2.70(1.66,4.44)	2.26(1.13,4.52)	0.021*
	CD4 > = 350 mm^3^	26(19.3)	105(39.2)	1	1	
History of TB co-infection	Yes	55(40.7)	45(16.8)	3.40(2.10,5.34)	2.59(1.31,5.13)	0.006*
	No	80(59.3)	223(83.2)	1	1	
History of ASM	Yes	26(19.3)	203(75.7)	0.08(0.05,0.13)	0.05(0.03,0.10)	< 0.001*
	No	109(80.7)	65(24.3)	1	1	
OI in the last 6 months	Yes	41(30.4)	33(12.3)	3.10(1.81,5.06)	3.06(1.49,6.28)	0.002*
	No	94(69.4)	235(87.7)	1	1	
HIV disclosure status	No	37(27.4)	44(16.4)	1.92(1.15,3.10)	2.15(0.98,4.69)	0.056
	Yes	98(72.6)	224(83.6)	1	1	

Variables with a p-value with” * “were variables significantly associated at p-value < 0.05

## Discussion

This study has identified that the odds of HIV-positive clients on first-line ART with an age greater or equal to 35 years were more than 3-fold increased for virologic failure when compared with those ages less than 35 years old during the last viral load test. This result is supported by the study conducted in the Amhara region, Waghimra Zone, in 2020 [4]. Similarly, it is supported by a study conducted in the United States of America in 2018 [8]. This might be due to the possibility of virologic failure increases as age increases because, as age increase individual’s immunity to prevent disease decrease and at the same time chronic and other disease increases which may expose individual for HIV virologic failure.

The result of this study showed that the odds of HIV-positive clients on first-line ART with single marital status were 3.77 fold higher for virologic failure when compared with married clients. Contradicting this result, a study conducted at Mettu Karl’s Referral Hospital and research conducted at Adigrat General Hospital showed no association between marital status and virologic failure among clients on ART [9], [10]. This is probably due to the fact that clients with single marital status have no support from their partner, may miss their drug, and may have poor treatment adherence.

The study also showed that the odds of clients with a baseline ART combination with AZT-based (AZT + 3TC + NVP) were more than four times higher to develop virologic failure compared to a d4T-based (d4T-3TC-NVP) combination ART regimen. This study contradicts a study conducted in the Amhara region in the Waghimra zone, which showed clients on AZT and the other two ART drug combinations at baseline had no association [11]. The result is supported by a study conducted in northeast Ethiopia, which showed the odds of clients on an AZT-based regimen at the time of the viral load test having a probability of 2.6-fold to develop virologic failure [12]. Furthermore, it is in line with research conducted in low- to middle-income countries that revealed over 80% of clients on first-line ART with an AZT/d4T-based regimen had predicted drug resistance, which is confirmed by virologic failure [13]. This is probably due to a viral mutation and the specific drug resistance of the zidovudine (AZT) drug [13]. 

The result of this study showed that the odds of a client with baseline CD4 less than 350 mm^3^ were found to be more than three times higher to face virologic failure compared to those with baseline CD4 greater than 350 mm^3^. The result is consistent with a study conducted at Nekemte Specialized Hospital [14]. This result is also similar to a study conducted in Ethiopia at Waghimra Amhara region in 2020 [4]. The finding of this study is also consistent with a study conducted in Thailand [15]. This is because as an individual’s HIV fails to suppress; CD4 count lowers number of HIV increase rapidly then client’s health deteriorates leading to treatment failure [6]. 

Furthermore, the odds of developing virological failure among clients with TB co-infection were more than threefold higher than those with no history of virologic failure. The result is consistent with the study conducted at Mettu Karl’s specialized hospital [16]. This result is concordant with research done at Adigrat General Hospital [10]. Similarly, this result is comparable to a study conducted at Dar Es Salaam, Tanzania, which revealed HIV-positive clients on first-line ART had odds of 2.1 to develop virologic failure compared to clients with no history of TB co-infection [17]. This may be because, like HIV, TB infection also attacks white blood cells, which leads to a synergistic effect with HIV to minimize CD4 count and facilitate HIV replication, which leads to HIV virologic failure. Additionally, clients with TB co-infection have pill burdens, anti-TB-ART drug-to-drug interactions, and poor HIV treatment outcomes.

The current study showed that clients who were enrolled in a six-month appointment spacing model to collect drugs and for clinical follow-up were associated with decreased virologic failure. However, this result is opposed to a study conducted at Debre Markos Town, which revealed virologic suppression decreased from a baseline of 99.22 to 96 after the implementation of ASM [18]. But the current result is supported by a cohort study done in Guinea, which revealed that ASM is associated with a reduction of 60% attrition from care and results in good viral suppression [19]. This is probably because ASM minimizes clients from missing their follow-up due to frequent transportation costs and distance barriers and allows them to work more since ASM visits only twice a year. So this may help clients with frustration, which enhances good treatment adherence and good viral suppression.

This study showed the odds of having an opportunistic infection other than TB in the last six months before the last viral load test during first-line ART were more than three times higher for HIV virologic failure. This result is also consistent with research conducted in Thailand [15]. This is probably when an opportunistic infection is present; the ability of the client to prevent HIV multiplication is decreased, and this leads to viral non-suppression. On the other hand, an opportunistic infection is a sign of a poor treatment outcome, which means there is an increased viral load [6]. 

The result of this study showed no significant association between DTG-based combinations of ART and virologic failure. However, the study conducted in the US Orlando immunology Centre shows patients on the new regimen with DTG combination as baseline treatment have an 82% chance of maintaining a viral load of less than 50 copies/mL following the switch [20]. 

### Strength

Relatively strong study design (case-control) Epi Info software was used to control data entry errors. Since the data were from a secondary data source, all adult clients in the multi-month dispense (MMD) model, like 6 months of ASM and 3 months of MMD, had been selected with equal chance.

### Limitations

The study also entirely depended on secondary data, which may affect the reliability of the data. Some variables, like personal behavior such as alcohol drinking and substance use, that could affect virologic failure were not included since no recorded data were available.

## Conclusion

This study showed that client age, marital status, baseline ART regimen, baseline CD4 cell count, history of Tb-co infection, and opportunistic infection in the last 6 months were factors associated with virologic failure among clients on first-line ART. Involvement in ASM was found to be protective of virologic failure.

## Abbreviations

AIDS: Acquired Immunodeficiency Syndrome
AOR: Adjusted Odds Ratio
ART: Anti-retroviral therapy
ASM: Appointment Spacing Model
AZT: Zidovudine
DSD: Differentiated service Delivery
DTG: Delutegravir
EFV: Efavirenz
HIV: Human Immunodeficiency Virus
LTFU: Lost To Follow Up
MMD: Multi-Month dispensing
NVP: Nevirapine
PREP: Pre-exposure prophylaxis
TB: Tuberculosis
TDF: Tenofovir
VIF: Variance Inflation Factor
VL: Viral Load
WHO: World Health Organization
